# Identification of Pheophytin a and Hydroxy Pheophytin a from Rang Chuet (*Thunbergia laurifolia* Linn.) as Potent NQO-1 Inducers in Liver Cells †

**DOI:** 10.3390/foods13101443

**Published:** 2024-05-07

**Authors:** Sreylak Moeurng, Kakanang Posridee, Anyanee Kamkaew, Siwatt Thaiudom, Anant Oonsivilai, Ratchadaporn Oonsivilai

**Affiliations:** 1 Department of Bioengineering, Faculty of Engineering, Royal University of Phnom Penh, Phnom Penh 12156, Cambodia; sreylakmoeurng@gmail.com; 2 Health and Wellness Research Group, School of Food Technology, Institute of Agricultural Technology, Suranaree University of Technology, Nakhon Ratchasima 30000, Thailand; posridee.ka@gmail.com (K.P.); thaiudom@sut.ac.th (S.T.); 3 School of Chemistry, Institute of Science, Suranaree University of Technology, Nakhon Ratchasima 30000, Thailand; anyanee@sut.ac.th; 4 School of Electrical Engineering, Institute of Engineering, Suranaree University of Technology, Nakhon Ratchasima 30000, Thailand

**Keywords:** *Thunbergia laurifolia* Linn. (Rang Chuet, RC), detoxification, liver cell lines (AML12, HepG2), bioactive compounds, pheophytin *a*, hydroxy pheophytin *a*, NQO-1 enzyme

## Abstract

*Thunbergia laurifolia* Linn. (Rang Chuet, RC), a Thai medicinal plant, possesses various bioactive compounds with potential health benefits. This study aimed to identify detoxifying compounds within RC crude extract. RC leaves were extracted using the Soxhlet method with chloroform. Total carotenoids, chlorophylls, extract yield, total phenolic contents (TPCs), and total flavonoid contents (TFCs) were measured. The extract’s composition was analyzed. Cytotoxicity and effects on the detoxification enzyme NQO-1 were assessed in liver cell lines (AML12 and HepG2) using MTT and NQO-1 assays, respectively. Bioactive fractions were identified using fractionation techniques and mass spectrometry (LC-MS). RC extract displayed significant levels of carotenoids (0.375 mg/g), chlorophylls (2.682 mg/g), and favorable yield (15.3%). TPC and TFC were 363.776 mg/g and 112.22 mg/g of extract, respectively. Analysis revealed phenolic acids (gallic acid, caffeic acid), flavonoid (apigenin), chlorophylls (chlorophylls *a*, *b*, pheophytin *a* and *b*), and lutein. Among the fractions, Fraction 3 (F3) exhibited the highest NQO-1 enzyme activity. F3 contained pheophytin *a* and hydroxy pheophytin *a*, confirmed by LC-MS (*m*/*z* 871.59^+^ [M + H]^+^ and 887.59^+^ [M + H]^+^). F3 significantly induced NQO-1 activity in both HepG2 (3.908-fold) and AML12 (1.99-fold) cells. This study identified F3 from RC extract as a promising fraction containing pheophytin *a* and hydroxy pheophytin *a*, responsible for inducing the detoxification enzyme NQO-1 in liver cells. These findings suggest RC’s potential for promoting detoxification.

## 1. Introduction

*Thunbergia laurifolia* Linn. (*Acanthaceae*), also known as Rang Chuet (RC) in Thai folk medicine, has a long history of use for centuries [1]. This plant serves as a natural source of various active compounds, including phenolic acids, flavonoids, iridoid glycosides, carotenoids, and chlorophyll [2,3]. Chemical analysis of Rang Chuet reveals the presence of specific constituents like caffeic acid, rosmarinic acid, apigenin, chlorophylls (*a* and *b*), pheophorbides (*a* and *b*), pheophytins (*a* and *b*), sterols, steroids, cardiac glycosides, gallic acid, protocatechuic acid, and lutein [1,2]. Additionally, the plant is a source of essential nutrients, containing protein, fat, ash, fiber, and carbohydrates [4].

Chromatographic techniques offer a well-established approach for isolating medicinal compounds. Plant leaf extracts comprise diverse bioactive compounds with varying polarities and molecular weights [5,6]. These polarity differences influence their interaction with the stationary phase (silica gel) through dipole interactions with hydroxyl groups [7]. The compounds with weaker affinity or lower absorption for the stationary phase elute faster compared to more polar substances, resulting in fractions [8]. Amarowicz et al. [9] explain that this separation relies on the principle that compounds with lower polarity move faster through the system.

In recent years, research has increasingly explored the potential of herbal plants as a source of natural therapeutic agents due to their abundance of phytochemicals with hepatoprotective and detoxifying properties [10,11]. Rang Chuet is a traditional Thai herb with documented pharmacological properties. Analysis using advanced techniques like high-performance liquid chromatography (HPLC) and nuclear magnetic resonance (NMR) has revealed the presence of bioactive compounds such as phenolic acids (gallic acid, caffeic acid) and flavonoids (rosmarinic acid and quercetin) within RC [2,3]. Additionally, reverse-phase HPLC has identified a group of pigments in RC, including chlorophylls, chlorophyll derivatives, and carotenoids [2]. The phytochemicals present in Rang Chuet exhibit potent antioxidant activity, with gallic acid demonstrating significant free radical scavenging abilities [12]. Furthermore, studies report the presence of microelements such as iron and selenium in the plant [13]. Notably, Suwanchaikasem et al. [13] successfully isolated rosmarinic acid from the Rang Chuet crude extract using absorbents such as Diaion HP20, silica gel, and Sephadex LH-20. This purified compound displayed remarkable antioxidant activity with an EC_50_ value of 2.71 µg/mL. Oonsivilai et al. [2] identified eight derivatives of total chlorophyll compounds in RC, including pheophorbide *a*, lutein, chlorophylls (*a* and *b*), and pheophytins (*a* and *b*). More recently, Junsi et al. [1] employed LC-MS for the specific identification of phenolic and flavonoid compounds within the plant.

Toxicity studies suggest that Rang Chuet (RC) may act as an antidote against poisoning by heavy metals, insecticides, and toxic drugs. *In vivo* research using Nile Tilapia fish demonstrated that RC leaf extract can induce lead excretion [14]. Ruangyuttikarn et al. [15] observed that while water extract of *Thunbergia laurifolia* Linn. could mitigate cadmium (Cd)-induced organ damage in the liver and kidney, blood and urine Cd levels remained unchanged. Similarly, Rana et al. [16] reported partial recovery of Cd-damaged kidney organs in rats treated with RC water crude extract. Notably, RC treatment (100 and 200 mg/kg body weight) led to a reduction in the expression of TNF-α, iNOS, and COX-2, all of which are inflammatory cytokines [16].

Detoxification, a critical physiological process, eliminates harmful substances, both internally generated (endogenous toxins) and those introduced from the external environment (xenobiotics) [17]. The liver, a vital human organ, houses this detoxification machinery, primarily through phase I and II metabolic reactions [17]. These reactions transform toxicants into water-soluble forms for efficient excretion. However, excessive exposure to toxins can overwhelm the liver’s capacity, leading to acute liver damage [18]. Biotransformation reactions are central to this process, with phase II enzymes playing a crucial role. These enzymes, including UDP-glucuronosyltransferases, sulfotransferases, N-acetyltransferases, glutathione S-transferases, and methyltransferases, facilitate the elimination of intermediate metabolites by adding functional groups and enhancing their water solubility [19]. Among these phase II enzymes, NAD(P)H quinone oxidoreductase-1 (NQO-1) stands out for its multifaceted functions. Beyond its role in detoxification, NQO-1 acts as a chaperone protein. Chaperones prevent non-specific aggregation of proteins by binding to unfolded or misfolded polypeptides, ensuring proper protein folding and function [20,21].

This study researches deeper into the detoxifying potential of RC. By separating the crude extract into fractions and analyzing them, this research aims to pinpoint the specific compounds responsible for this beneficial effect.

## 2. Materials and Methods

### 2.1. Rang Chuet Extraction

*Thunbergia laurifolia* leaves were collected in March 2021 from the Non-toxic Agriculture Cooperative under the Royal Initiative of His Majesty the King (NACR) The people of a neighborhood’s Development Study Center in Nakhon Ratchasima Province, Thailand. The herbarium voucher specimen (Ratchadaporn 001) was prepared and deposited at the School of Food Technology, Suranaree University of Technology. Five grams of powdered *Thunbergia laurifolia* (Rang Chuet) leaves were loaded into a cellulose thimble (Whatman, GE Healthcare, Rotherham, UK) for Soxhlet extraction. Chloroform (200 mL) was used as the solvent. The extraction process was carried out at 50 °C for 5 h. The collected extract was then evaporated to dryness, yielding the crude extract which was subsequently stored in a freezer [22].

### 2.2. Phytochemical Profiling

#### 2.2.1. Total Phenolic Contents

The total phenolic content of the extracts was determined using the Folin-Ciocalteu method on microplates. Briefly, 100 µL of Folin-Ciocalteu reagent was added to 20 µL of extract solution (0.5 mg/mL). The mixture was then incubated with 80 µL of a 7.5% (*w*/*v*) Na_2_CO_3_ solution for 30 min at room temperature in the dark. After incubation, the absorbance was measured at 765 nm. A standard curve prepared using gallic acid solutions (0, 50, 100, 250, 500, and 750 µg/mL) allowed for the quantification of the phenolic content, expressed as milligrams of gallic acid equivalents (GAEs) per gram of extract [23,24].

#### 2.2.2. Total Flavonoid Contents

The total flavonoid content was determined using an aluminum chloride method with quercetin as the standard. Briefly, 250 µL of the plant extract was mixed with 100 µL of distilled water. Then, 10 µL of a 5% NaNO_2_ solution was added, and the mixture was incubated for 5 min. Subsequently, 10 µL of a 10% AlCl_3_ solution was added, followed by another incubation for 6 min. After incubation, the absorbance was measured at 510 nm using a microplate reader. A standard curve was constructed using quercetin solutions at various concentrations (0, 50, 100, 250, 500, and 750 µg/mL) to quantify the flavonoid content, expressed as quercetin equivalents (QEs) per gram of extract [24,25,26].

#### 2.2.3. Total Chlorophylls and Carotenoids

Following a previously reported method [27], 0.2 g of sample powder was extracted with 15 mL of 80% acetone. The absorbance of the extract was measured at 663.2 nm, 646.8 nm, and 470 nm using a spectrophotometer. These measurements were then employed to calculate the concentration of chlorophyll and carotenoids according to established equations [1,2,3,4].

chla (µg/mL) = 12.25 × A663.2 − 2.79 × A646.8(1)

chlb (µg/mL) = 21.50 × A646.8 − 5.1 × A663.2(2)

TChl (µg/mL) = chla + chlb(3)

Car (µg/mL) = (1000 × A470 − 1.8 × chla − 85.02 × chlb)/198(4)

The results expressed in milligram per gram of raw material (mg/g RM) [28].

#### 2.2.4. Chlorophyll Profiling

A previously reported method [2,29] was used for chlorophyll profiling by reverse-phase HPLC. Briefly, the chloroform crude extract (25 mg/mL) and standard solutions (50–500 µg/mL) were filtered through a 0.22 µm nylon membrane prior to injection onto a GRACE Vydac C18 column (L250 × ID 4.6 mm, 201TP54). A gradient mobile phase system was employed, consisting of (A) methanol: water: ammonium acetate (73:25:2, *v*/*v*) and (B) ethyl acetate. The flow rate was maintained at 1.0 mL/min with a total run time of 30 min. Detection was performed across a broad wavelength range (250–600 nm) to capture chlorophyll pigments. Calibration curves were constructed using standard solutions (50–500 µg/mL) for quantification.

The analysis compared the extract profile to commercially available standards (Sigma-Aldrich Co., St. Louis, MO, USA) of lutein, pheophytin a, pheophytin b, chlorophyll a, and chlorophyll b. Pheophytin a and b standards were prepared following a published method [2] by converting chlorophyll a and b, respectively. Briefly, 1 mg of chlorophyll a or b was dissolved in 10 mL of acetone, followed by the addition of 400 µL of 0.1 N HCl to a 5 mL aliquot. The solution was then kept at room temperature until the color changed from green to olive-brown, indicating pheophytin formation. Subsequently, the solvents were evaporated using N_2_ gas, the residue was redissolved in acetone, filtered through a 0.22 µm PTFE membrane, and stored at −80 °C.

#### 2.2.5. Thin-Layer Chromatography

Thin-layer chromatography (TLC) 60F254 (Sigma-Aldrich, Steinheim, Germany) was employed to select an appropriate solvent system for subsequent silica gel column chromatography 60 µm (Sigma-Aldrich, Steinheim, Germany). The crude extract was spotted onto a TLC plate as a band with dimensions of 1 cm width and 5 cm length. The plate was then developed using a solvent system composed of hexane and ethyl acetate (70:30, *v*/*v*). The separation of spots on the TLC plate was monitored to identify a suitable solvent system for further purification.

#### 2.2.6. Flask Column Chromatography

A glass chromatography column (7 × 50 cm) was pre-equilibrated with pure hexane and packed with silica gel. Briefly, the column was rinsed with hexane, and a small wad of cotton was placed at the bottom to retain the stationary phase. Silica gel powder was then slurred in hexane and carefully poured into the column, allowing for homogeneous packing. The crude extract was adsorbed onto a portion of silica gel before loading onto the top of the column. Elution was performed using a gradient mobile phase system, starting with a mixture of hexane and ethyl acetate (80:20, *v*/*v*) and increasing the polarity by gradually increasing the ethyl acetate content (e.g., 70:30, *v*/*v*). The eluted Fractions (Fs) were collected and concentrated by evaporation. The initial identification of compounds within these fractions was achieved using thin-layer chromatography (TLC).

### 2.3. Cell Culture

#### 2.3.1. Cytotoxicity of Human Liver Hepatoma (HepG2) and Alpha Mouse Liver 12 (AML12) Cell Lines

Cell culture, human liver hepatoma (HepG2) cell lines and alpha mouse liver 12 (AML12) cell lines used in this study were purchased from the American Type Culture Collection (ATCC^®^ HB-8065^TM^, CRL-2254^TM^, Manassas, VA, USA). Cell viability assays were employed to assess the cytotoxicity of the crude extract and its fractions on HepG2 and AML12 cell lines. Cells were seeded at a density of 10^4^ cells/well in 96-well plates and incubated for 24 h before treatment. The crude extract and fractions were applied at various concentrations (0.031–5 mg/mL) for another 24 h. The MTT assay was then performed to evaluate cell viability. Briefly, MTT reagent was added to the cells and incubated for 3 h. After washing, the formazan crystals were dissolved in DMSO, and the absorbance was measured at 590 nm using a microplate reader [30]. Wells without cells were used as blanks and were subtracted as background from each sample. Cell viability was expressed as a percentage of the control values. Cell viability (%) was calculated based on the equation established by Adewusia et al. [31]. The IC_50_ value, representing the concentration that inhibits 50% cell growth, was also determined.

Cell Viability (%) = (Absorbance of cell treatments × 100)/Absorbance of control cells

IC_50_ = (50 − b)/a (5)

The dose-response was constructed for finding y = ax + b.

#### 2.3.2. NAD(P)H Quinone Oxidoreductase-1 (NOQ-1)

NQO-1 enzyme activity was determined using a commercially available NQO-1 activity assay kit (AB184867, Abcam, Bangkok, Thailand) following the manufacturer’s instructions and considering previously established cell viability data. Briefly, after a 24-h treatment period, the culture medium was removed, and cells were washed with PBS. Cell lysates were then prepared by adding 100 µL of 1× extraction buffer and incubating the cells on ice for 15 min. Following centrifugation at 18,000× *g* for 20 min at 4 °C, the supernatant was collected. A 50 µL aliquot of the diluted supernatant (1:2) was loaded in duplicate wells for the reaction buffer and inhibitor controls. The plate was immediately read at a wavelength of 440 nm to measure the development of yellow color, indicative of NQO-1 activity. NQO-1 enzyme activity for each fraction was determined in triplicate and expressed as fold change.

#### 2.3.3. Compound Identification by Thin-Layer Chromatography and Liquid Chromatography-Mass Spectrometry (LC-MS)

Analysis of the TLC plates revealed distinct spot patterns for the crude extract (RC) and its fractions. The first spot, likely corresponding to carotenes, exhibited a yellow color, like Fraction 1 (F1). Fraction 7 (F7) also displayed a spot indicative of lutein. Meanwhile, F2 contained a single spot consistent with pheophytin b, while F3 displayed a spot for pheophytin a. Fractions F4, F5, and F6 displayed spot patterns characteristic of chlorophylls. These observations aligned well with the findings of Oonsivilai et al. [2] regarding pigment profiles in RC extracts. To further characterize the fractions, the content of total phenolics, total flavonoids, and total chlorophylls was determined.

To identify the NQO-1 activity-inducing compound(s) within Fraction 3 (F3), further analysis was performed using high-performance liquid chromatography (HPLC) coupled with tandem mass spectrometry (LC-MS/MS). Fraction 3 (F3) was re-dissolved in HPLC-grade methanol, filtered, and stored at −80 °C for injection. An aliquot of 10 µL was directly injected into an LC-MS/QTOF (Thermo Scientific Ultimate 3000, Bangkok, Thailand) mass spectrometer. The separation was achieved using an isocratic mobile phase consisting of 5% (A): 95% (B). Solvent A was water with 0.1% formic acid, while solvent B was acetonitrile with 0.1% formic acid. In the positive ionization mode, the mass spectrometer was operated with a scan range of *m*/*z* 50–1700, an electrospray ionization (ESI) source voltage of 4.5 kV, and a source temperature of 180 °C.

### 2.4. Statistical Analysis

All experiments were performed in triplicate, and mean values (on a dry basis) with standard deviations are reported. The experimental data were analyzed using an analysis of variance (ANOVA). SPSS^®^ software version 17 (SPSS Inc., Chicago, IL, USA) was used to perform all statistical calculations.

## 3. Results

Table 1 showed the quantified levels of TPC, TFC, and TChl in each fraction obtained from the RC crude extract. Fractions 1 (F1) and 7 (F7) displayed the highest TPC (8.749 ± 0.60 and 11.095 ± 0.02 mg GAE/g extract, respectively). Interestingly, both F1 and F7 also exhibited similar TChl levels (0.635 ± 0.0255 and 0.62 ± 0.0288 mg/g extract, respectively). This suggests the presence of phenolic compounds with hydroxyl groups capable of redox reactions and contributing to antioxidant activity (lutein in F7) alongside pigments like carotenes (F1) which lack such functionalities. Carotenes, with their characteristic isoprenoid structure rich in carbon and hydrogen, are known for their free radical scavenging abilities [32].

Fraction 2 (F2) displayed the highest TChl content (10.70 ± 0.418 mg/g extract), followed by F5, F6, F4, and F2. Statistical analysis (Table 1) revealed significant differences (*p* < 0.05) in the content of most phytochemicals across the fractions. Notably, all fractions displayed low levels of TFC, suggesting the potential presence of polar flavonoid compounds that might have remained adsorbed on the silica gel during column chromatography.

### 3.1. Cell Viability and Cytotoxicity in Human Liver Hepatoma (HepG2) and Alpha Mouse Liver 12 (AML12) Cell Lines 

**Figure 1:**
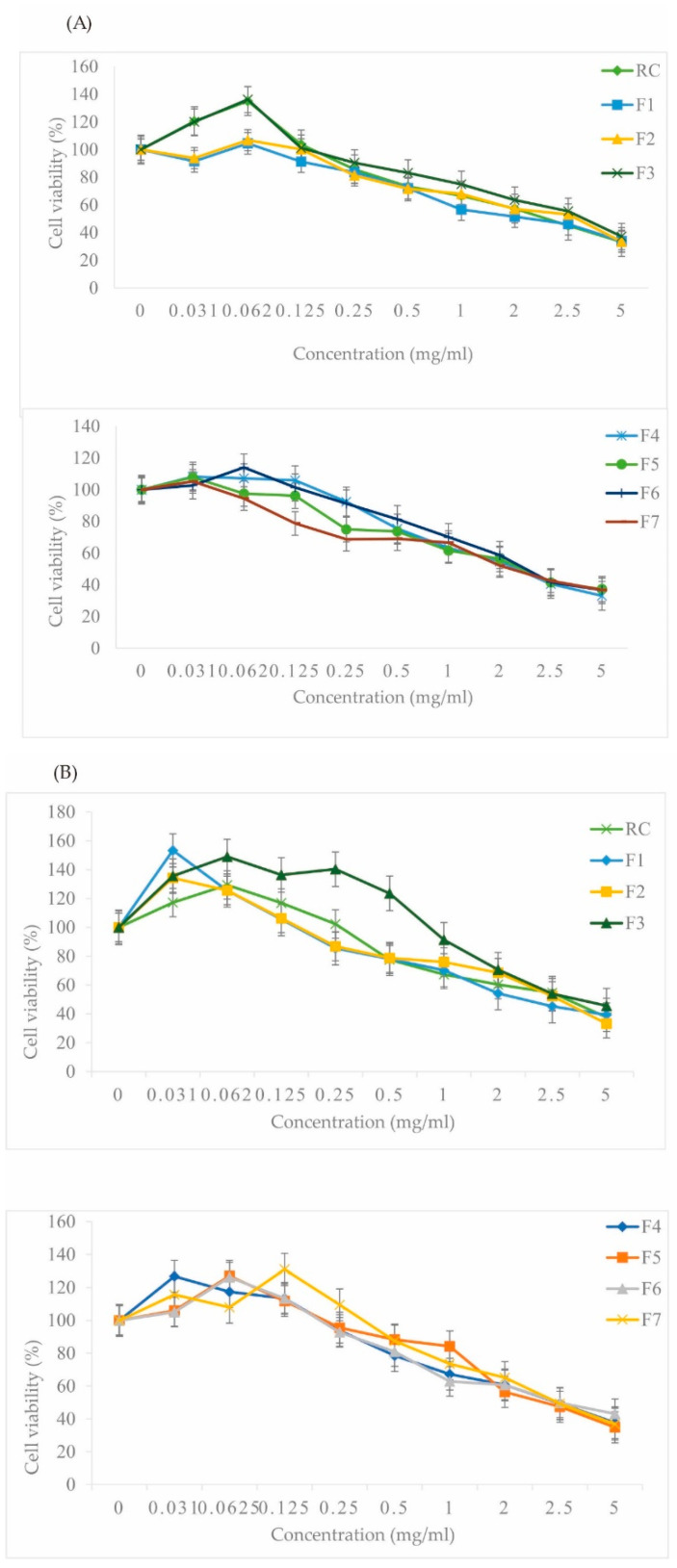
The viability of AML12 (**A**) and HepG2 (**B**) cell lines following treatment with RC crude extract fractions (F1–F7) is presented. The data represent the percentage of viable cells relative to the control group, expressed as mean ± SD (*n* = 3 independent experiments).

Cell viability and cytotoxicity of the RC crude extract and its fractions were evaluated using the MTT assay [33]. This assay measures the activity of cellular oxidoreductase enzymes, which convert a yellow tetrazolium dye to a purple formazan product in metabolically active cells. RC crude extract and fractions (ranging from 5 to 0.031 mg/mL) were applied to HepG2 and AML12 cells. As shown in Figure 1, cell viability remained above 50% in both cell lines at concentrations exceeding 2 mg/mL, indicating minimal cytotoxicity at these concentrations.

Cytotoxicity assays are widely used to assess the therapeutic potential and safety of drugs by evaluating their effect on cell viability [33]. In this study, the IC_50_ values of the RC crude extract and its fractions were determined and found to be above 2 mg/mL for all but Fraction 1 (F1) in the AML12 cell line (Table 2). High IC_50_ values indicate a low level of cytotoxicity, suggesting the chemoprotective potential of most RC constituents [34]. While F1 exhibited an IC_50_ value slightly below 2 mg/mL in AML12 cells, the difference was not statistically significant (*p* < 0.05). Furthermore, the dose-response curves demonstrate minimal toxicity to mammalian cells at all tested concentrations, with high cell viability maintained. These findings provide preliminary data for further exploration in future pharmaceutical trials.

### 3.2. Specific Enzyme Activities NAD(P)H Quinone Oxidoreductase-1 (NQO-1)

NQO-1, encoded by the NQO-1 gene on chromosome 16q22.1, is a key enzyme involved in cellular defense mechanisms [35]. It functions by neutralizing electrophiles and oxidants, protecting mammalian cells from harmful compounds. Notably, NQO-1 plays a crucial role in stabilizing the tumor suppressor protein p53, further contributing to its anti-cancer properties [36]. Furthermore, NQO-1 is a vital component of phase II detoxification. This enzyme catalyzes the reduction in toxic substrates, including both endogenous and environmental quinones, rendering them harmless [37]. Through a double-displacement reaction involving NAD(P)H as a cofactor, NQO-1 converts these toxicants into hydroquinones, effectively removing them from the cell [38].

Table 3 summarizes the NQO-1 enzyme activity in AML12 and HepG2 cell lines following a 24-h exposure to RC crude extract and its fractions. The RC crude extract induced a specific NQO-1 activity increase of approximately 1.387 ± 0.073-fold compared to the control in AML12 cells. Notably, Fraction 3 (F3) elicited the highest enzyme activity induction (1.99 ± 0.047-fold). Fractions F4, F2, F5, and F6 also displayed significant increases in NQO-1 activity (1.66 ± 0.45, 1.472 ± 0.064, 1.420 ± 0.299, and 1.392 ± 0.041-fold, respectively). In contrast, F1 demonstrated the lowest induction (1.174 ± 0.263-fold), while F7 induced a moderate increase (1.445 ± 0.122-fold). Although most differences in specific NQO-1 activity compared to controls in AML12 cells were not statistically significant, Fractions F3 and F4 exhibited significant activity increases (*p* < 0.05).

Interestingly, the effects on NQO-1 activity were more pronounced in HepG2 cells. Fraction 3 (F3) again displayed the strongest induction (3.908 ± 0.124-fold), followed by F4, F5, F6, F2, F7, and F1 (2.67 ± 0.187, 2.562 ± 0.083, 2.332 ± 0.15, 2.033 ± 0.079, 2.281 ± 0.034, and 1.701 ± 0.074-fold, respectively). All fractions significantly increased NQO-1 activity compared to controls in HepG2 cells.

These findings suggest that bioactive compounds in the RC extract may induce NQO-1 gene expression, leading to increased enzyme activity. Additionally, the NQO-1 activity levels in AML12 cells were significantly different from those in HepG2 cells for most treatment groups (Figure 1), indicating potential cell-type specific effects.

**Figure 2:**
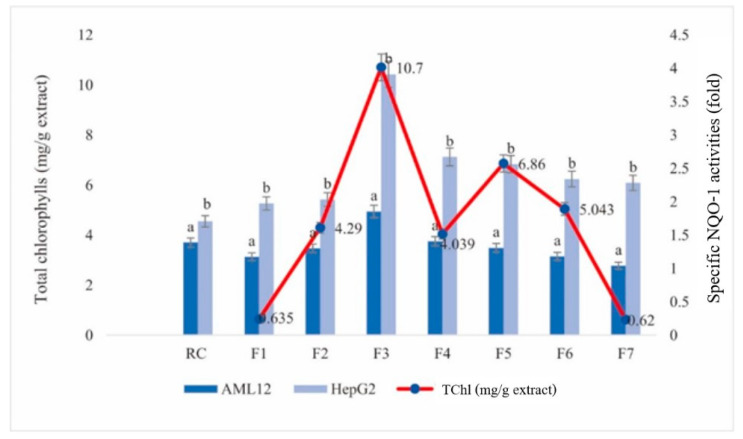
The specific NQO-1 enzyme activities in AML12 and HepG2 cell lines and the quantities of total chlorophylls in each fraction. Noted: The same subscript in each bar graph represented a non-significant difference (*p* < 0.05).

The lower NQO-1 enzyme induction observed in F1 and F7 compared to other fractions might be attributed to differences in their bioactive compound profiles (Table 3). Notably, F3, which exhibited the highest NQO-1 activity induction, also displayed the highest total chlorophyll content (10.7 mg/g extract of TChl) (Table 1). This suggests a potential link between certain bioactive compounds, particularly chlorophylls, and NQO-1 enzyme expression. The initial TLC analysis (result now shown) revealed that F1 and F7 primarily consisted of carotenoids, while F2 contained pheophytin b and F3 contained pheophytin a. Fractions F4, F5, and F6 were identified as containing chlorophylls. These findings provide preliminary insights into the potential bioactive components responsible for the observed NQO-1 induction effects. The observed correlation between NQO-1 enzyme activity and total chlorophyll (TChl) content suggests a potential link between these factors (Table 3, Figure 2). Notably, Fraction 3 (F3) displayed the highest NQO-1 induction and the highest TChl content (10.7 ± 0.418 mg/g extract). This finding raises the possibility that certain bioactive compounds within the RC extract, particularly chlorophylls and their derivatives, might contribute to NQO-1 enzyme expression.

The chemical characteristics of chlorophylls and their derivatives, such as the presence of double bonds, could play a role in this observed effect [39]. These functionalities might enhance their ability or the ability of their metabolites to react with thiol groups (R-SH) found in the cysteine amino acid. Cysteine plays a crucial role in enzyme expression, and these interactions could potentially influence NQO-1 activity levels. The observed correlation between NQO-1 enzyme activity and total chlorophyll (TChl) content warrants further investigation (Table 3, Figure 2). Notably, Fraction 3 (F3) exhibited the strongest NQO-1 induction and the highest TChl content (10.7 ± 0.418 mg/g extract). This finding suggests a potential link between specific bioactive compounds in the RC extract, particularly chlorophylls and their derivatives, and NQO-1 enzyme expression.

### 3.3. Compounds Identification

**Figure 3:**
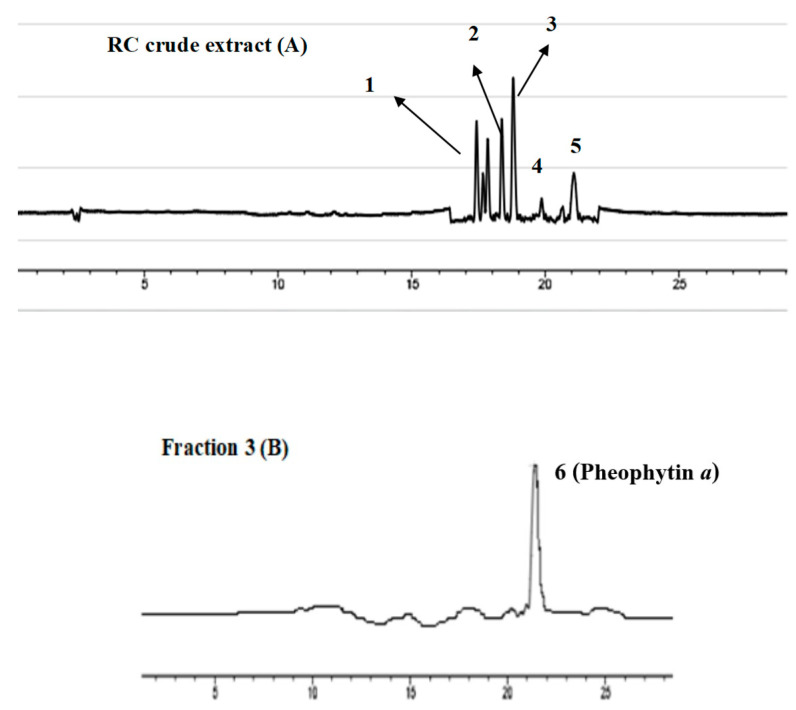
The chromatograph represented the compound in RC crude extract and F3, and HPLC showed peaks: (1) lutein, (2) chlorophyll *b*, (3) chlorophyll *a*, (4) pheophytin *b*, (5) pheophytin *a*. Peak separation was detected at 600 nm; pheophytin *a* released the peak at a retention time of 21.5 min.

Pheophytin *a* was found in Fraction 3 (F3) on the primary identifying TLC plate, the fraction was also verified by HPLC and liquid chromatography-mass spectrometry (LC-MS). Figure 3A showed the chromatogram of RC chloroform crude extract and Figure 3B was a Fraction 3 (F3) measured by high-performance chromatography. The presence of pheophytin *a* in Fraction 3 (F3), initially identified using the TLC plate, was further confirmed by HPLC and liquid chromatography-mass spectrometry (LC-MS) analyses. Figure 3A depicts the HPLC chromatogram of the RC chloroform crude extract, while Figure 3B shows the chromatogram of Fraction 3 (F3). These results provide complementary evidence for the presence of pheophytin *a* within this fraction.

**Figure 4:**
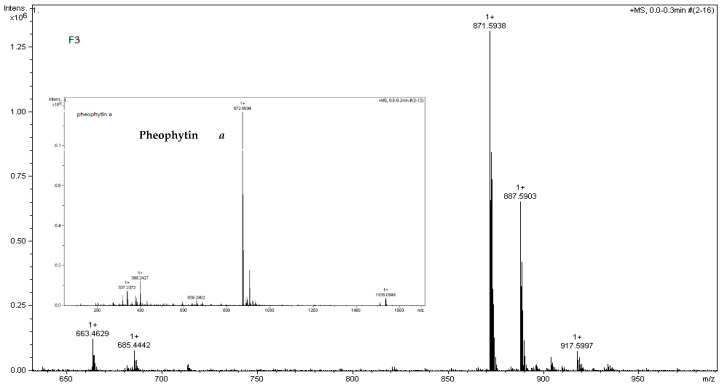
The result of LC-MS spectra generated from Fraction 3 (F3) was similar to the chemical standard. F3 was directly injected 10 µL into LC-MS/QTOF. The condition of MS was held at the scan range *m*/*z* 50–1700.

The two peaks found in F3, pheophytin *a* and hydroxy pheophytin *a*, were shown at the *m*/*z* 871.59^+^ [M + H]^+^ and 887.59^+^ [M + H]^+^ (Table 4, Figure 4). Jerz et al. [39] reported the *m*/*z* 887.5697 value for [M + H]^+^ was monoisotopic of C_55_H_74_N_4_O_6_, namely, a 13^2^-hydroxy pheophytin *a.*

The high NQO-1 enzyme induction observed in Fraction 3 (F3) (Table 3) could be attributed, at least in part, to the presence of pheophytin a and its hydroxylated derivative, as confirmed by HPLC and LC-MS (Figure 4). These compounds might contribute to the detoxification activity of the fraction. Additionally, Fraction 3 (F3) displayed the highest total chlorophyll (TChl) content (Figure 3) and some level of total phenolic content (TPC) and total flavonoid content (TFC). This suggests that the combined presence of various bioactive compounds within F3, including chlorophylls, pheophytins, and potentially phenolic and flavonoid components, may contribute to its overall biological activity, including its ability to induce NQO-1 enzyme expression.

## 4. Discussion

Chloroform extraction offers a unique advantage in capturing a diverse range of bioactive compounds from RC extracts, including TPC, TFC, TChl, and carotenes, as compared to other solvents [2]. While water extracts may yield higher levels of TPC, as reported by Junsi et al. [1] and Oonsivilai et al. [2], these might not necessarily translate to superior biological activity. Nawaz et al. [40] observed that despite lower TPC and TFC content, non-polar solvent extracts often exhibit greater biological efficacy.

In this study, the chloroform extract displayed a yield of 15.3%, with a moderate level of total chlorophyll (2.68 ± 0.125 mg/g RM) and carotenoids (0.375 ± 0.032 mg/g RM). Interestingly, all fractions exhibited low levels of TFC. This could be attributed to the presence of polar flavonoid compounds that might have remained adsorbed on the silica gel during the column chromatography process [41]. Since flavonoids are typically a subgroup of phenolics, their lower abundance compared to TPC further suggests the potential adsorption of these polar compounds on the silica gel.

The separation of bioactive compounds during column chromatography relies on their interactions with the stationary and mobile phases [42]. In our study, a C18 column, characterized by its high hydrophobicity, was employed. This type of column typically elutes less polar compounds, such as C-7 carbons, before more polar ones like C-15 carbons [43]. Consequently, compounds with higher retention times will appear later in the chromatogram.

The findings on the cytotoxicity of RC crude extract fractions in HepG2 cells align with previous studies [44,45]. These reports demonstrated that RC extract exhibits minimal toxicity at concentrations ranging from 0.01 to 2 mg/mL in HepG2 cells. Additionally, Preechasuk et al. [28] reported no adverse effects associated with RC extract consumption at doses of 9–12 mg/day. These observations suggest potential cytoprotective properties of the bioactive compounds present in RC.

The observed differences in NQO-1 enzyme activity between AML12 and HepG2 cells could be attributed to their distinct cellular metabolism and signaling pathways. Normal cell proliferation and enzyme production are tightly regulated by factors like p53 tumor suppressor genes, which receive signals from neighboring cells [46]. Conversely, cancer cells exhibit uncontrolled proliferation, often due to a disregard for these regulatory signals. This abnormal cellular behavior can lead to the overproduction of potentially abnormal or dysfunctional proteins [47,48]. Notably, Fang et al. [48] demonstrated that HepG2 cells expressed significantly higher NQO-1 activity (1782 ± 72 nmoL/mg extract) compared to Caco2 cells (10 ± 1.0 nmoL/mg extract) under similar treatment conditions with tanshinone IIA. This finding further supports the concept of cell-type specific regulation of NQO-1 activity.

Initial TLC analysis revealed that Fractions F1 and F7 primarily consisted of carotenoids, while F2 contained pheophytin b and F3 contained pheophytin a. Fractions F4, F5, and F6 were identified as chlorophyll a, chlorophyll b, and an unknown compound, respectively. Interestingly, F3, the fraction exhibiting the highest NQO-1 enzyme induction, also contained pheophytin a. This finding aligns with previous studies by Fahey et al. [41] and Oonsivilai et al. [2], who demonstrated the ability of chlorophylls to induce NQO-1 activity in different cell lines. Furthermore, Oonsivilai et al. [2] reported that an acetone extract, rich in chlorophylls and their derivatives, displayed significantly higher NQO-1 activity compared to water and ethanol extracts, which were richer in phenolics and flavonoids. These observations suggest a potential role for chlorophylls and their derivatives in NQO-1 enzyme expression.

Mechanistically, Zhang et al. [49] proposed that chlorophylls and chlorophyllin may induce NQO-1 and heme oxygenase-1 (HO-1) by promoting the accumulation of the transcription factor Nrf2 and activating the PI3K/Akt signaling pathway. Additionally, studies by Balder et al. [50] suggest a potential protective effect of dietary chlorophylls against colon cancer. Taken together, these findings support the notion that chlorophylls and their derivatives might play a role in NQO-1 induction and potentially contribute to the anticancer properties of RC extract.

While chlorophylls are recognized for their antioxidant and detoxification properties, some studies suggest lower activity compared to pheophytins [51]. Notably, pheophytins, lacking the central metal atom present in chlorophylls, exhibit diverse biological effects [4]. These include antioxidant activity, modulation of genotoxic effects, inhibition of cytochrome P450 enzymes, cell cycle arrest, and induction of phase II detoxification enzymes like NQO-1 and glutathione S-transferase.

## 5. Conclusions

In conclusion, *Thunbergia laurifolia* Linn. (RC) leaves emerge as a promising source of health-promoting bioactive compounds. Chloroform extraction yielded a rich profile of potentially chemoprotective phytochemicals, including phenolics, flavonoids, carotenoids, and chlorophylls. Notably, Fraction 3 (F3), enriched in pheophytin *a*, exhibited the strongest NQO-1 enzyme induction in liver cell lines. This finding suggests a potential link between pheophytin *a* and NQO-1 activity, warranting further investigation into the underlying mechanisms.

## Figures and Tables

**Table 1:** The polyphenol content and total chlorophyll in each fraction.

Fraction	TPC (mg GAE/g Extract)	TFC (mg QE/g Extract)	TChl (mg/g Extract)
F1	8.749 ± 0.608 ^a^	2.361 ± 0.133 ^a^	0.635 ± 0255 ^a^
F2	4.474 ± 0.094 ^b^	1.548 ± 0.249 ^b^	4.29 ± 0.171 ^b^
F3	5.463 ± 0.31 ^c^	1.71 ± 0.095 ^c^	10.70 ± 0.418 ^c^
F4	3.23 ± 0.197 ^d^	0.631 ± 0.133 ^d^	4.039 ± 0.095 ^b^
F5	5.858 ± 0.718 ^e^	1.613 ± 0.305 ^e^	6.86 ± 0.455 ^d^
F6	4.937 ± 0.01 ^f^	0.361 ± 0.064 ^f^	5.043 ± 0025 ^e^
F7	11.095 ± 0.02 ^g^	1.069 ± 0.205 ^g^	0.62 ± 0.288 ^a^

Note: Data are presented in means ± SD, the different letters in the same column represent significant differences (*p* < 0.05).

**Table 2:** Cytotoxicity of RC crude extract and fraction in HepG2 and AML12.

Active Compounds/Cell Line	IC_50_ (mg/mL)	
	AML12	HepG2
RC extract	2.042 ± 0.067 ^a^	2.676 ± 0.095 ^a^
F1	2.056 ± 0.142 ^a^	2.377 ± 0.11 ^b^
F2	2.556 ± 0.045 ^b^	2.818 ± 0.109 ^c^
F3	3.139 ± 0.096 ^c^	3.583 ± 0.13 ^d^
F4	2.25 ± 0.078 ^d^	2.546 ± 0.078 ^e^
F5	2.228 ± 0.070 ^d^	2.583 ± 0.12 ^e^
F6	2.288 ± 0.079 ^d^	2.836 ± 0.14 ^c^
F7	2.07 ± 0.12 ^a^	2.67 ± 0.13 ^a^

Note: IC_50_ value calculated in means ± SD in the same row with the different subscripts are significantly different (*p* < 0.05).

**Table 3:** Induction of NAD(P)H quinone oxidoreductase-1 (NQO-1) activity in HepG2 and AML12 cells line.

Fraction	NQO-1 AML12	NQO-1 HepG2
Control	1 ^a^	1 ^a^
RC extract	1.387 ± 0.073 ^a^	1.705 ± 0.142 ^b^
F1	1.174 ± 0.263 ^a^	1.973 ± 0.0742 ^c^
F2	1.472 ± 0.064 ^a^	2.033 ± 0.079 ^c^
F3	1.99 ± 0.047 ^b^	3.908 ± 0.124 ^d^
F4	1.66 ± 0.45 ^b^	2.67 ± 0.187 ^e^
F5	1.420 ± 0.299 ^a^	2.562 ± 0.083 ^e^
F6	1.392 ± 0.041 ^a^	2.339 ± 0.15 ^f^
F7	1.445 ± 0.122 ^a^	2.281 ± 0.034 ^f^

Noted: Values of NQO-1 activities were calculated in means ± SD, the same subscript in each column represented a non-significant difference (*p* < 0.05).

**Table 4:** MS spectral data of chlorophylls and their derivation.

No.	Compound	[M + H]^+^ (Online)	[M + H]^+^ (Reported)
1	Chlorophyll *a*	893	-
2	Chlorophyll *b*	907	-
3	Hydroxy pheophytin *b*	901	-
4	Pheophytin *b*	885	-
5	Pheophytin *a*	871.5	871.59
6	Hydroxy pheophytin *a*	887.4	887.59

## Data Availability

The original contributions presented in the study are included in the article, further inquiries can be directed to the corresponding authors.
